# Gene Expression Profiling Reveals the Shared and Distinct Transcriptional Signatures in Human Lung Epithelial Cells Infected With SARS-CoV-2, MERS-CoV, or SARS-CoV: Potential Implications in Cardiovascular Complications of COVID-19

**DOI:** 10.3389/fcvm.2020.623012

**Published:** 2021-01-15

**Authors:** Prabhash Kumar Jha, Aatira Vijay, Arda Halu, Shizuka Uchida, Masanori Aikawa

**Affiliations:** ^1^Center for Excellence in Vascular Biology, Brigham and Women's Hospital, Harvard Medical School, Boston, MA, United States; ^2^Department of Cardiovascular and Metabolic Sciences, Lerner Research Institute, Cleveland Clinic, Cleveland, OH, United States; ^3^Center for Interdisciplinary Cardiovascular Sciences, Brigham and Women's Hospital, Harvard Medical School, Boston, MA, United States; ^4^Channing Division of Network Medicine, Brigham and Women's Hospital, Harvard Medical School, Boston, MA, United States; ^5^Department of Clinical Medicine, Center for RNA Medicine, Aalborg University, Copenhagen, Denmark

**Keywords:** SARS-CoV-2, SARS-CoV, MERS-CoV, COVID-19 and transcriptome analysis, cardiovasclar disease

## Abstract

Severe acute respiratory syndrome coronavirus 2 (SARS-CoV-2) is the causative virus for the current global pandemic known as coronavirus disease 2019 (COVID-19). SARS-CoV-2 belongs to the family of single-stranded RNA viruses known as coronaviruses, including the MERS-CoV and SARS-CoV that cause Middle East respiratory syndrome (MERS) and severe acute respiratory syndrome (SARS), respectively. These coronaviruses are associated in the way that they cause mild to severe upper respiratory tract illness. This study has used an unbiased analysis of publicly available gene expression datasets from Gene Expression Omnibus to understand the shared and unique transcriptional signatures of human lung epithelial cells infected with SARS-CoV-2 relative to MERS-CoV or SARS-CoV. A major goal was to discover unique cellular responses to SARS-CoV-2 among these three coronaviruses. Analyzing differentially expressed genes (DEGs) shared by the three datasets led to a set of 17 genes, suggesting the lower expression of genes related to acute inflammatory response (TNF, IL32, IL1A, CXCL1, and CXCL3) in SARS-CoV-2. This subdued transcriptional response to SARS-CoV-2 may cause prolonged viral replication, leading to severe lung damage. Downstream analysis of unique DEGs of SARS-CoV-2 infection revealed changes in genes related to apoptosis (NRP1, FOXO1, TP53INP1, CSF2, and NLRP1), coagulation (F3, PROS1, ITGB3, and TFPI2), and vascular function (VAV3, TYMP, TCF4, and NR2F2), which may contribute to more systemic cardiovascular complications of COVID-19 than MERS and SARS. The study has uncovered a novel set of transcriptomic signatures unique to SARS-CoV-2 infection and shared by three coronaviruses, which may guide the initial efforts in the development of prognostic or therapeutic tools for COVID-19.

## Introduction

The novel severe acute respiratory syndrome coronavirus 2 (SARS-CoV-2) belongs to the Coronaviridae family of viruses (coronaviruses) and is responsible for the coronavirus disease 2019 (COVID-19) pandemic (1). Along with its other accomplices, Middle East respiratory syndrome coronavirus (MERS-CoV) and severe acute respiratory syndrome coronavirus (SARS-CoV), SARS-CoV-2 can jump species barrier followed by human-to-human transmission via droplet infection. In late December 2019, initial reports suggested the origin of SARS-CoV-2 in a seafood and wild animal trading market in Wuhan, China (2). To date, the pandemic has caused more than 83 million infections and more than 1.8 million deaths worldwide (https://www.worldometers.info/coronavirus/). SARS-CoV-2 leads to more cardiovascular complications than do MERS-CoV and SARS-CoV; however, what causes these major differences remains obscure (3, 4).

The initial genome identification of SARS-CoV-2 suggested that it has a ~80% similarity with SARS-CoV and 96% identical to a bat coronavirus; however, there are differences in its pathogenicity and host response (2). The virus nucleic acid shedding patterns in both symptomatic and asymptomatic patients of SARS-CoV-2 are similar, which explains the transmission potential of otherwise asymptomatic carriers (5). In contrast, the viral burden in the upper respiratory tract in SARS-CoV infection peaks at around 10 days after the initial exposure (6). On the contrary to SARS-CoV-2, viral load in MERS-CoV–infected individuals peak at week 2 of the onset of infection (7). This suggests the difference in the virulence and host response of these three strains.

Upon entry, next steps are viral replication, amplification, and spread in the host, which largely depend on similarities and/or uniquenesses in transcriptional signature of these viruses. Patients with SARS-CoV-2 manifest a few different but aggravated symptoms, particularly major cardiovascular complications, from SARS-CoV and MERS-CoV, which may be attributable to the difference in their transcriptional signatures. Reports of aggravated blood coagulation in COVID-19 patients suggested the mechanism of prominent elevation of d-dimer and fibrin/fibrinogen degradation products (8). Higher mortality rate is reported in COVID-19 patients with thromboembolic events (9), and treatment with anticoagulant-heparin has improved prognosis (10).

The present comparative analysis has determined key differences in transcriptional changes in lung epithelial cells induced by these virus strains. To further examine transcriptional responses of SARS-CoV-2 and other two coronaviruses, we analyzed a comprehensive map of lung epithelial cells infected with these three coronaviruses and explored pathological host responses unique to SARS-CoV-2. Our findings may help to understand potential mechanisms by which SARS-CoV-2 causes more cardiovascular complications than do two other coronaviruses and to establish molecular bases for the development of therapies against COVID-19.

## Methods

### RNA Sequencing and Microarray Analysis of Gene Expression Omnibus Datasets

**Figure 1:**
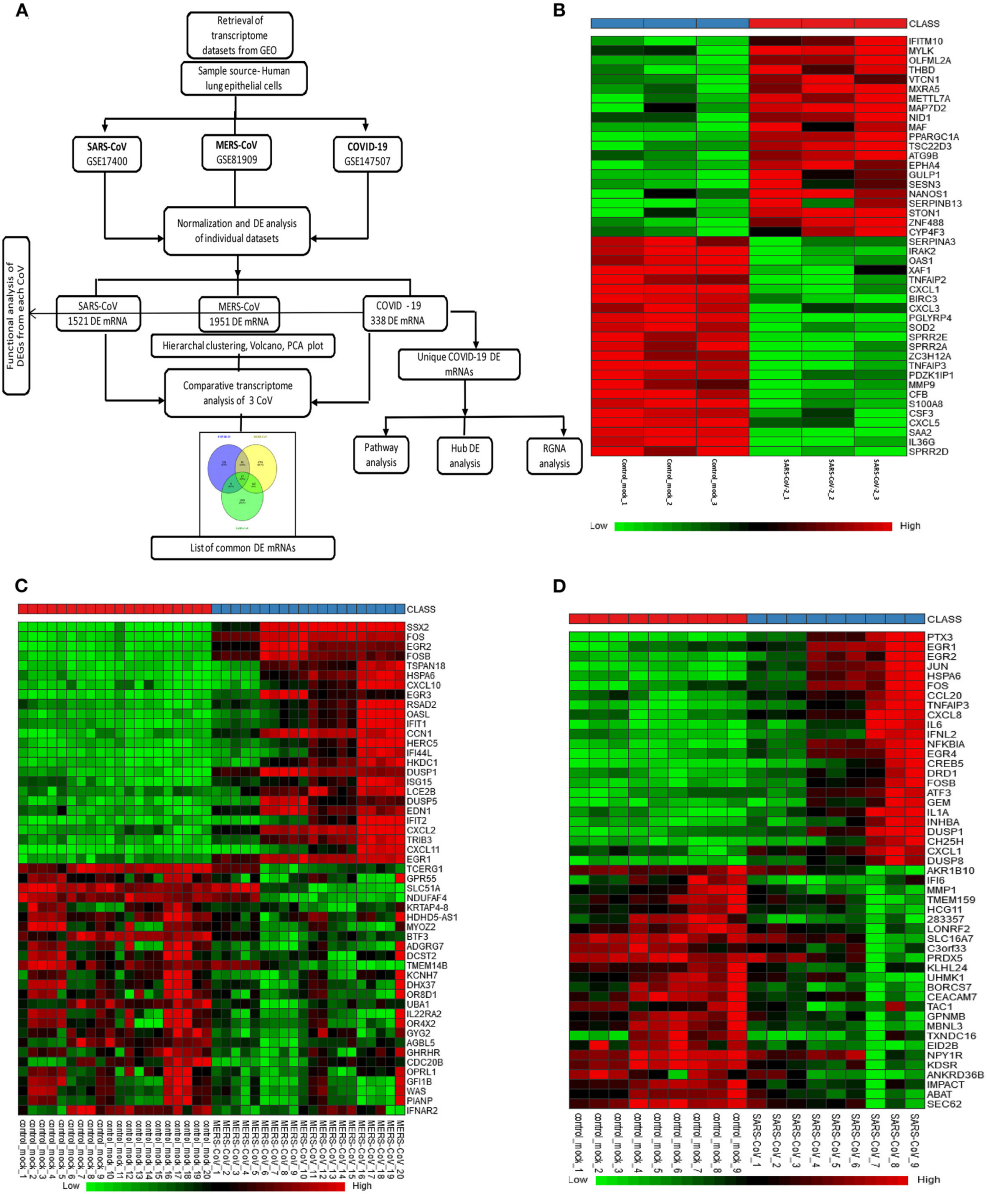
Gene expression profiles of the differentially expressed genes (DEGs) in human lung epithelial cells infected with coronaviruses. **(A)** Workflow of gene expression analysis. Selection process of eligible datasets for transcriptome analysis was based on datasets generated from infection of human lung epithelial cell in culture with SARS-CoV-2, MERS-CoV, or SARS-CoV. SARS-CoV-2, severe acute respiratory syndrome coronavirus 2; CoV, coronavirus; MERS-CoV, Middle East respiratory syndrome coronavirus; SARS-CoV, severe acute respiratory syndrome coronavirus and GEO, Gene Expression Omnibus. **(B–D)** Heatmaps of expression profiles for the top 25 increased and 25 decreased DEGs obtained from RNA-seq data analysis. Clustering of selected genes on the heatmap was performed by hierarchical clustering algorithm using Euclidean distance measure. **(B)** SARS-CoV-2, **(C)** MERS-CoV, and **(D)** SARS-CoV.

Figure 1A depicts the workflow of gene expression analysis. For differential gene expression analysis of SARS-CoV-2 infection, raw expression counts were downloaded from Gene Expression Omnibus (GEO) accession number GSE147507 (11). RNA sequencing (RNAseq) dataset was generated on Illumina Nextseq 500 platform. The raw read counts were normalized by log2 transformation, before and after normalization box plot; principal component analysis (PCA) and density plot are shown in Supplementary Figure 1. Using INMEX tool that employs DESeq (12, 13), differential expression analysis was performed and differentially expressed genes (DEGs) were characterized for each sample with adjusted *p* < 0.05 [false discovery rate (FDR) corrected by Benjamini–Hochberg method]. GSE81909, the dataset we used for analysis of MERS-CoV, was generated on Agilent-Whole Human Genome Microarray 4x44K G4112. After downloading the raw read counts from GEO, we normalized the dataset using variance-stabilizing normalization followed by quantile normalization (14). Before and after normalization box plot, PCA and density plot are shown in Supplementary Figure 2. Similarly, we downloaded raw read counts of GSE17400 (15) for analysis of SARS-CoV infection. This dataset was generated on Affymetrix Human Genome U133 plus 2.0 Array. After normalization of dataset using variance-stabilizing normalization followed by quantile normalization (Supplementary Figure 3), both microarray datasets, GSE81909 and GSE17400, were subjected to DEGs analysis using LIMMA algorithm (16). DEGs were characterized for each sample with adjusted *p* < 0.05 (FDR corrected by Benjamini–Hochberg method). Heatmap visualization of a subset of 25 overexpressed and underexpressed genes was constructed using heatmap.2 from the gplot package in R. Volcano plots were constructed using custom scripts in R, and PCA was performed on log2 fold-change values using PMA package in R (17) (Supplementary Figure 4). It is worthwhile mentioning that all three datasets used in this study are collected from different laboratories and using different cell lines, as well as experimental techniques (e.g., microarrays, RNAseq). We selected RNAseq dataset for SARS-CoV-2 as there were especially no microarray datasets on SARS-CoV-2 in humans. Therefore, we did take the present analysis strategy of comparing each dataset with its internal control to call the DEGs for each coronavirus infection. Further, we compared the three sets of DEGs to find the shared and unique genes between coronavirus infections; we applied this strategy to minimize data variabilities (e.g., operator and platform biases). GSE147507 was generated in primary human lung epithelium (NHBE); GSE81909 was generated in human airway epithelial cells, whereas GSE17400 was generated in human bronchial epithelial cells. Supplementary Table 1 provides detailed information of each dataset and sequencing/microarray platform used.

### Functional Gene Set Enrichment Analysis of DEGs

To discern the implication of DEGs called from transcriptome analysis of coronavirus infection in lung epithelial cells, we performed a functional analysis using the EnrichR platform (18). This web-based software product evaluates significantly enriched pathways/terms in an input gene list with the help of its extensive gene set libraries, which includes Gene Ontology (GO) (19) and various pathway analysis libraries such as Kyoto Encyclopedia of Genes and Genomes (KEGG) pathway, Reactome pathway, wikipathway, Panther, and Biocarta. We retrieved tables of enriched pathways from each database and prepared a comprehensive table of most significant pathways for each coronavirus infection based on the adjusted *p* value (ranking derived from Fisher exact test for gene sets) significance.

### Common DEGs Analysis Between Coronaviruses

**Figure 2:**
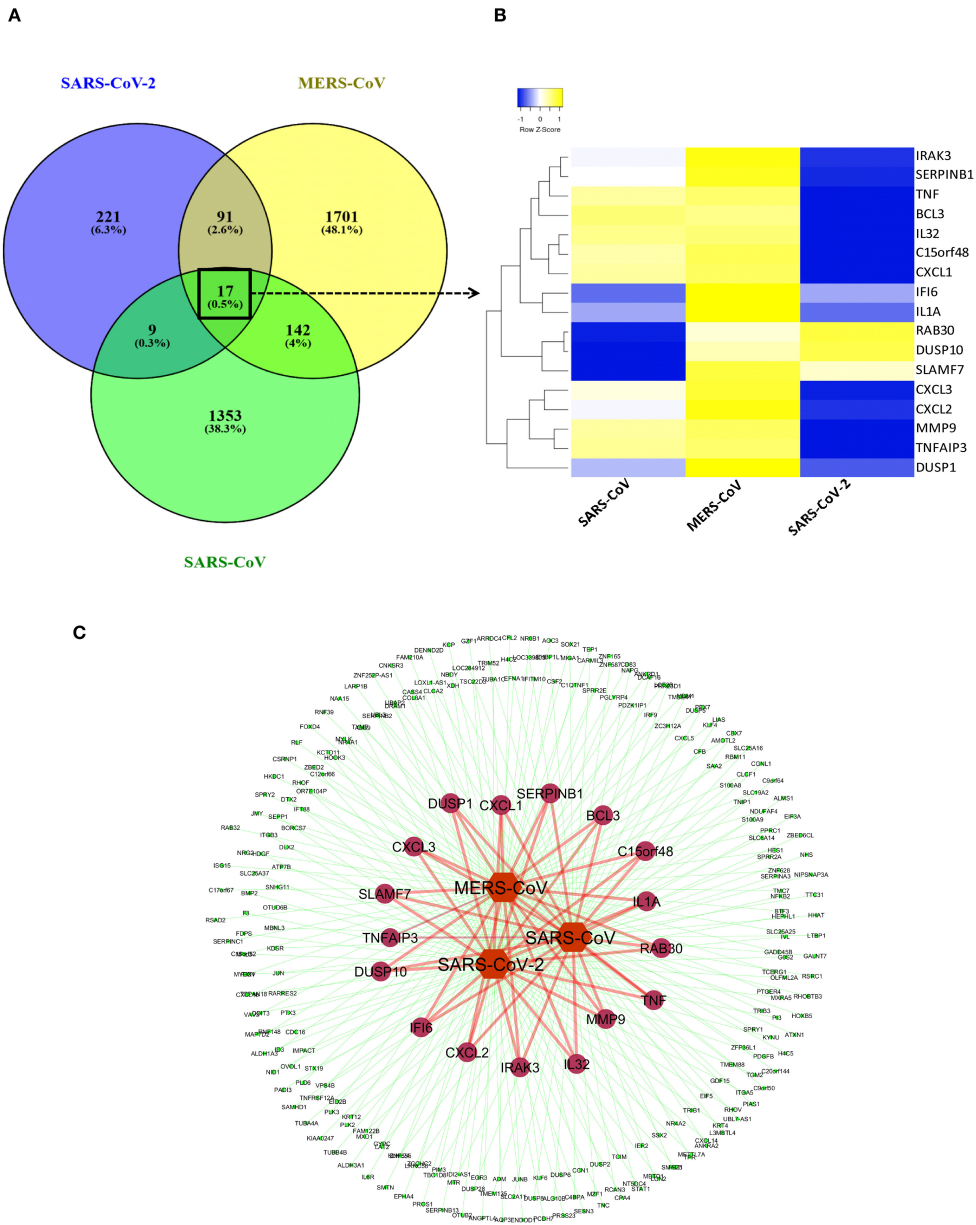
Shared transcriptional signatures between the three coronaviruses. **(A)** Venn diagram representing the shared and unique DEGs portion between three coronaviruses. **(B)** Heatmap representation of expression profiles for the common DEGs between coronaviruses. Clustering of selected genes on the heatmap was performed by hierarchical clustering algorithm using Euclidean distance measure. Expression scale: blue (low expression) to yellow (high expression). **(C)** coronavirus–gene network representing the shared and unique DEGs. Network was created between coronaviruses and their top 100 DEGs on cytoscape platform. Network core represents the coronaviruses (source nodes) and gene (target nodes). Inner circles of genes in the network are the shared ones, while outer circle genes are unique to each coronavirus.

We created a coronavirus–gene network for better visualization of the shared genes between the coronaviruses using Cytoscape software (20). The network was generated by utilizing the list of DEGs from three coronaviruses studies in which coronaviruses are the source nodes; genes are the target nodes, and the connections between them are the edges in the network. The network core represents the coronaviruses, whereas the inner-circle genes in the network are the shared ones, and outer-circle genes are unique to each coronavirus (Figure 2C). A Venn diagram representing the shared and unique DEG portion between three coronaviruses was generated using VENNY 2.1 tool (https://bioinfogp.cnb.csic.es/tools/venny/index.html). A heatmap represents the expression profiles for common DEGs between coronaviruses. Clustering of selected genes on the heatmap was performed by hierarchical clustering algorithm utilizing Euclidean distance measure.

### Pathway Clustering and Network-Based Hub Gene Analysis

For visualization and interpretation of the biological relevance of unique DEGs to SARS-CoV-2 DEGs, Cytoscape v3.1 plug-in was used for analysis. Biological pathway clustering analysis was done using BinGO (21). BinGO analyzes GO terms and functional groups association within the biological networks. We performed biological pathway clustering analysis to see collective function of these genes. The size of a node is proportional to the number of targets in the biological process category. The color represents enrichment significance—the deeper the color on a color scale, the higher the enrichment significance. Hub gene network analysis was performed using NetworkAnalyst (13), which created a protein–protein interaction (PPI) network by integrating the InnateDB interactome with the original seed of 221 DEGs. This tool supports integrative analysis of gene expression data through statistical, visual, and network-based analysis approaches by taking the advantage of common functions for network topology and module analysis approaches. Briefly, the complete list of unique DEGs from SARS-CoV-2 was uploaded into the web-based server of NetworkAnalyst. Network construction was restricted to contain all the original seed proteins in order to visualize the connections. To help identify highly interconnected hub nodes, topological measures (e.g., degree and betweenness centrality) were used. Expression of the genes was considered as the network feature, where red-colored nodes are genes with increased expression, green nodes are genes with decreased expression, and gray nodes are genes not expressed in our data.

### Expression2Kinases Analysis of Regulatory Gene Networks

ChEA is a comprehensive databases of kinases and transcription factors (22), and it is used in background of Expression2Kinases (X2K) (23), the tool we used to understand the upstream regulatory molecules of DEGs in SARS-CoV-2 infection. The 10 most significant transcription factors and kinases were extracted based on Fisher exact test *p* value enrichment scoring. We downloaded the “.graphml” file generated from the analysis to create and visualize regulatory network on Cytoscape environment. This ensures that the protein network obtained during network expansion is properly connected by automatically increasing the path length, so that there are more intermediate proteins used to connect the transcription factors. In the network, a yellow node represents intermediate proteins in the PPI regulatory network. Node size represents the significance of protein based on adjusted *p* value; the bigger the nodes size, the higher the significance value.

### Statistical Analyses

For differential expression analysis of SARS-CoV-2 dataset GSE147507, read counts were subjected to differential expression analysis using INMEX, which utilizes the Rpackage DESeq (13). Genes with adjusted *p* < 0.05 were considered significant. The *p* value adjustment for multiple comparisons was done by the Benjamini–Hochberg method. For MERS-CoV-GSE81909 and SARS-CoV-GSE17400, differential expression analysis was performed with LIMMA algorithm for each dataset, independently using adjusted *p* < 0.05, based on the FDR using the Benjamini–Hochberg method and moderated *t* test. Significantly enriched GO terms were identified using hypergeometric tests, and *p* ≤ 0.05 was applied as a cutoff for statistical significance.

## Results

### Selection of Eligible Gene Expression Datasets for Coronavirus Infection in Human Lung Epithelial Cells

We selected three studies from the GEO accession numbers: GSE147507 for SARS-CoV-2, GSE81909 for MERS-CoV, and GSE17400 for SARS-CoV. The search was limited to transcriptome data generated in human lung epithelial cells. Figure 1A depicts the overall workflow of the analysis in this study. A total of 3/3, 20/20, and 9/9 control/infected cell culture replicates SARS-CoV-2, MERS-CoV, and SARS-CoV, respectively, were used in this analysis. GSE147507 dataset was RNAseq data and generated on Illumina Nextseq 500, and we utilized only six samples (GSM4432378, GSM4432379, GSM4432380, GSM4432381, GSM4432382, and GSM4432383); these were independent biological triplicates of primary human lung epithelium (NHBE), which were mock treated or infected with SARS-CoV-2 (USA-WA1/2020). Of note, the other two datasets were generated by microarray using Affymetrix Human Genome U133A series (GSE17400-SARS-CoV) and Agilent-014850 Whole Human Genome Microarray 4x44K G4112F (GSE81909-MERS-CoV). Sample sources of all three datasets were of human lung epithelial cells and primary lung cells infected with coronaviruses. Supplementary Table 1 provides detailed information of each dataset and sequencing/microarray platform used.

### Analysis of Differentially Expressed Genes (DEGs) in the SARS-CoV-2 Dataset Led to Perturbation of Inflammatory, Coagulation, and Apoptotic Pathways

In SARS-CoV-2–infected dataset (GSE147507), we identified a total of 338 DEGs with adjusted *p* < 0.05 (Supplementary Dataset 1). Among these 338 DEGs, 92 genes increased, and 246 decreased. Figure 1B depicts the heatmap of expression of top significant DEGs among the samples. Table 1 lists the top 20 increased and decreased DEGs from our analysis of SARS-CoV-2 infection. Interferon (IFN)–induced transmembrane protein 10 (*IFITM10*), C-X-C motif chemokine ligand 14 (*CXCL14*), and myosin light chain kinase (*MYLK*) were among the most significantly increased genes, while small proline-rich protein 2D (*SPRR2D*), interleukin 36 gamma (*IL36G*), and serum amyloid A2 (*SAA2*) were the most decreased genes in our analysis of SARS-CoV-2–infected lung epithelial cells compared to mock controls. When these DEGs were subjected to the analysis of overrepresented biological pathways and enriched terms, several pathways related to inflammation, apoptosis, blood coagulation, and lung fibrosis were enriched (Table 2). Enriched terms and biological pathways were significantly overrepresented in the gene list if they showed an adjusted *p* < 0.05. DEGs from SARS-CoV-2 infection were associated with the KEGG pathways such as IL-17 signaling pathway (hsa04657) with database overlap of 21/93 (which means of 93 genes associated with this pathway reported in KEGG, 21 are present among our DEGs) and adjusted *p* = 1.24E-15 and TNF signaling pathway (hsa04668) with database overlap of 19/110 and adjusted *p* = 4.76E-12. Besides, other databases resulted in enrichment of pathways including blood coagulation (P00011) with overlap of 7/38 and adjusted *p* = 1.67E-04, apoptosis signaling pathway (P00006) with overlap of 7/102 and adjusted *p* = 0.038822, and lung fibrosis (WP3624) with overlap of 11/63 and adjusted *p* = 4.53E-07, among others.

**Table 1:** Top 20 DEGs identified in the SARS-CoV-2 analysis.

**Gene ID**	**EntrezID**	**Gene Name**	**baseMean**	**P.Value**	**adj.P.Val**	**logFC**
**Increased DEGs**						
IFITM10	402778	Interferon-induced transmembrane protein 10	194.45	9.90E-13	2.60E-10	1.0377
CXCL14	9547	C-X-C motif chemokine ligand 14	100.64	4.25E-08	6.47E-06	0.8338
MYLK	4638	Myosin light chain kinase	90.676	4.45E-08	6.71E-06	0.83025
OLFML2A	169611	Olfactomedin like 2A	413.24	4.58E-12	1.16E-09	0.79462
THBD	7056	Thrombomodulin	404.62	8.33E-11	1.96E-08	0.79168
VTCN1	79679	V-set domain containing T cell activation inhibitor 1	182.21	4.56E-08	6.79E-06	0.76269
MXRA5	25878	Matrix remodeling associated 5	721.64	1.21E-11	2.96E-09	0.76162
METTL7A	25840	Methyltransferase like 7A	149.49	9.17E-08	1.34E-05	0.74126
MAP7D2	256714	MAP7 domain containing 2	54.277	5.84E-06	0.000547	0.69934
GPNMB	10457	Glycoprotein nmb	2057.8	8.18E-11	1.96E-08	0.68713
**Decreased DEGs**						
SPRR2D	6703	Small proline rich protein 2D	365.31	1.79E-53	8.02E-50	−2.1217
IL36G	56300	Interleukin 36 gamma	271.41	6.98E-57	4.68E-53	−2.0691
SAA2	6289	Serum amyloid A2	575.93	3.57E-81	4.79E-77	−2.0679
CXCL5	6374	C-X-C motif chemokine ligand 5	104.68	2.18E-35	2.44E-32	−1.8864
MX1	4599	MX dynamin like GTPase 1	427.77	4.07E-37	4.96E-34	−1.7731
CSF3	1440	Colony-stimulating factor 3	68.054	2.84E-28	1.81E-25	−1.6959
S100A8	6279	S100 calcium-binding protein A8	1707.4	4.04E-52	1.35E-48	−1.6127
ICAM1	3383	Intercellular adhesion molecule 1	1885	8.73E-45	1.95E-41	−1.5713
CFB	629	Complement factor B	789.4	2.86E-44	5.47E-41	−1.5634
MMP9	4318	Matrix metallopeptidase 9	318.36	6.38E-26	3.42E-23	−1.5215

*Genes were ranked based on the log fold change and adjusted p value (<0.05). The corresponding p values are adjusted, based on the false discovery rate using the Benjamini–Hochberg procedure*.

**Table 2:** Top enriched terms identified by functional analysis of the DEGs from SARS-CoV-2–infected human lung epithelial cells.

**Enrichment terms**	**Pathway/term ID**	**Overlap**	**GSEA library**	**Adjusted *p* value**
IL-17 signaling pathway	hsa04657	21/93	KEGG	1.24E-15
TNF signaling pathway	hsa04668	19/110	KEGG	4.76E-12
Signal transduction through IL1R	h il1rPathway	06/36	Biocarta	0.006706
NF-κB activation by nontypeable *Haemophilus influenzae*	h nthiPathway	05/29	Biocarta	0.013491
Plasminogen-activating cascade	P00050	07/15	Panther	2.37E-07
Blood coagulation	P00011	07/38	Panther	1.67E-04
Apoptosis signaling pathway	P00006	07/102	Panther	0.038822
Hemostasis	R-HSA-109582	27/552	Reactome	1.91E-04
Platelet degranulation	R-HSA-114608	11/105	Reactome	2.81E-04
Lung fibrosis	WP3624	11/63	Wikipathway	4.53E-07

*Overlap: indicates the number of hits from the meta-analysis compared to each curated gene set library. Gene set functional analysis was performed using extended libraries of the EnrichR tool. Enriched terms and pathways were ranked based on the p value. KEGG, Kyoto Encyclopedia of Genes and Genomes; GO, Gene Ontology Biological Process; GSEA, Gene Set Enrichment Analysis*.

### Identification of DEG Signature in MERS-CoV– or SARS-CoV–Infected Human Lung Epithelial Cells

In the case of MERS-CoV dataset, GSE81909, there are a total of 1,951 DEGs with adjusted *p* < 0.05 (Supplementary Dataset 2). Among these 1,951 DEGs, 1,120 genes increased, and 831 decreased. The microarray analysis of GSE17400 for SARS-CoV infection resulted in a total of 1,521 DEGs with adjusted *p* < 0.05 (Supplementary Dataset 3). Among these 1,521 DEGs, 475 increased, and 1,046 decreased. Figures 1C,D depict the heatmap of expression of top significant DEGs among the samples for MERS-CoV and SARS-CoV, respectively. As shown in Supplementary Table 2, SSX family member 2 (*SSX2*), fos proto-oncogene, AP-1 transcription factor subunit (*FOS*), and early growth response 1 (*EGR1*) were among the most significantly increased genes, whereas transcription elongation regulator 1 (*TCERG1*), G protein–coupled receptor 55 (*GPR55*), and casein kappa (*CSN3*) were the most decreased genes in our analysis of MERS-CoV–infected lung epithelial cells compared to controls. Similarly, Supplementary Table 3 shows that pentraxin 3 (*PTX3*), early growth response 1 (*EGR1*), and EGR2 were among the most significantly increased genes, whereas aldo-keto reductase family 1 member B10 (*AKR1B10*), IFN-α-inducible protein 6 (*IFI6*), and matrix metallopeptidase 1 (*MMP1*) are the most decreased genes in our analysis of SARS-CoV–infected lung epithelial cells compared to mock controls. When these DEGs from both MERS-CoV and SARS-CoV were subjected to the analysis of overrepresented biological pathways and enriched terms, several pathways related to inflammation and apoptosis were commonly enriched (Supplementary Tables 4, 5).

### Muted Expression of Acute Inflammatory Genes Was Observed in the SARS-CoV-2 When Compared to MERS-CoV-2 and SARS-CoV

Previous studies revealed that lung epithelial cells, dendritic cells, and macrophages all express cytokines to some extent during major viral infection causing cytokine storm. However, little is known about the situation in COVID-19. Earlier studies showed IFN-γ-related cytokine storm in SARS-CoV infection, whereas MERS-CoV infection had delayed induction of proinflammatory cytokines and suppression of innate antiviral response. It is crucial to identify the primary source of the cytokine storm in response to SARS-CoV-2 infection and the underlying virological mechanisms. Our analysis of shared DEGs between three coronaviruses resulted in 17 shared DEGs (Figure 2A), among which most genes are related to acute inflammation. Figure 2B indicates that the expression levels of these genes were lower in SARS-CoV-2 when compared with the other two coronaviruses. Our identification of a muted transcriptional response to SARS-CoV-2 supports a model in which initial failure to rapidly respond to infection results in prolonged viral replication and subsequent recruitment of proinflammatory cells as the infection progresses to induce alveolar damage in COVID-19. Table 3 depicts the expression value of DEGs shared in all three coronaviruses. It is evident that the expression of critical acute inflammatory genes including TNF-α-induced protein 3 (*TNFAIP3*), C-X-C motif chemokine ligand 1 (*CXCL1*), and *TNF* was lower in the SARS-CoV-2 dataset compared to two other coronaviruses. These results may suggest that lung epithelial cells do not directly contribute to the cytokine storm during COVID-19 and that other immune cells appear to participate in this process.

**Table 3:** Shared DEGs between coronaviruses.

		**COVID-19**		**MERS-CoV**		**SARS-CoV**	
**Symbols**	**Name**	**logFC**	**adj.P.Val**	**logFC**	**adj.P.Val**	**logFC**	**adj.P.Val**
TNFAIP3	TNF-α-induced protein 3	−1.4397	6.31E-48	1.3062	8.28E-07	1.0224	0.010541
CXCL1	C-X-C motif chemokine ligand 1	−1.2721	1.39E-38	0.88054	5.05E-07	0.54891	0.005485
C15orf48	chromosome 15 open reading frame 48	−1.1055	3.28E-28	0.62913	0.024618	0.27721	0.048513
IL32	Interleukin 32	−1.0607	1.44E-21	0.55427	6.13E-06	0.4451	0.021275
CXCL2	C-X-C motif chemokine ligand 2	−1.1072	1.87E-16	1.9732	6.51E-13	0.31032	0.025261
CXCL3	C-X-C motif chemokine ligand 3	−1.2936	4.47E-15	1.5534	1.66E-07	0.49265	0.006207
IL1A	Interleukin 1 alpha	−0.90381	3.18E-13	0.79049	3.99E-05	−0.63885	0.029208
SERPINB1	Serpin family B member 1	−0.65532	1.12E-09	0.2583	0.001145	−0.16797	0.034799
DUSP10	Dual specificity phosphatase 10	0.49169	0.000102	0.43159	0.017097	0.24686	0.014842
TNF	Tumor necrosis factor	−0.72256	0.000217	0.32484	0.003589	0.19034	0.039241
BCL3	BCL3 transcription coactivator	−0.57674	0.0004	0.45737	0.001256	0.53122	0.007329
IFI6	Interferon α-inducible protein 6	−0.49295	0.009535	0.4471	0.02091	−0.63885	0.029208
RAB30	“RAB30, member RAS oncogene family”	0.4949	0.023868	0.38751	0.000627	0.1745	0.048354
IRAK3	Interleukin 1 receptor associated kinase 3	−0.50297	0.024857	0.27287	0.034926	−0.14418	0.034042
SLAMF7	SLAM family member 7	0.44487	0.02517	0.72071	1.59E-07	−0.23004	0.028654
DUSP1	Dual specificity phosphatase 1	−0.34689	0.025301	2.1214	1.58E-20	0.24686	0.014842

*Expression values of the shared genes from each analysis. The corresponding p values are adjusted, based on the false discovery rate using the Benjamini–Hochberg procedure*.

### SARS-CoV-2 Elicits Suppressed Type I IFN Response and Activation of Apoptotic Gene Signature

**Figure 3:**
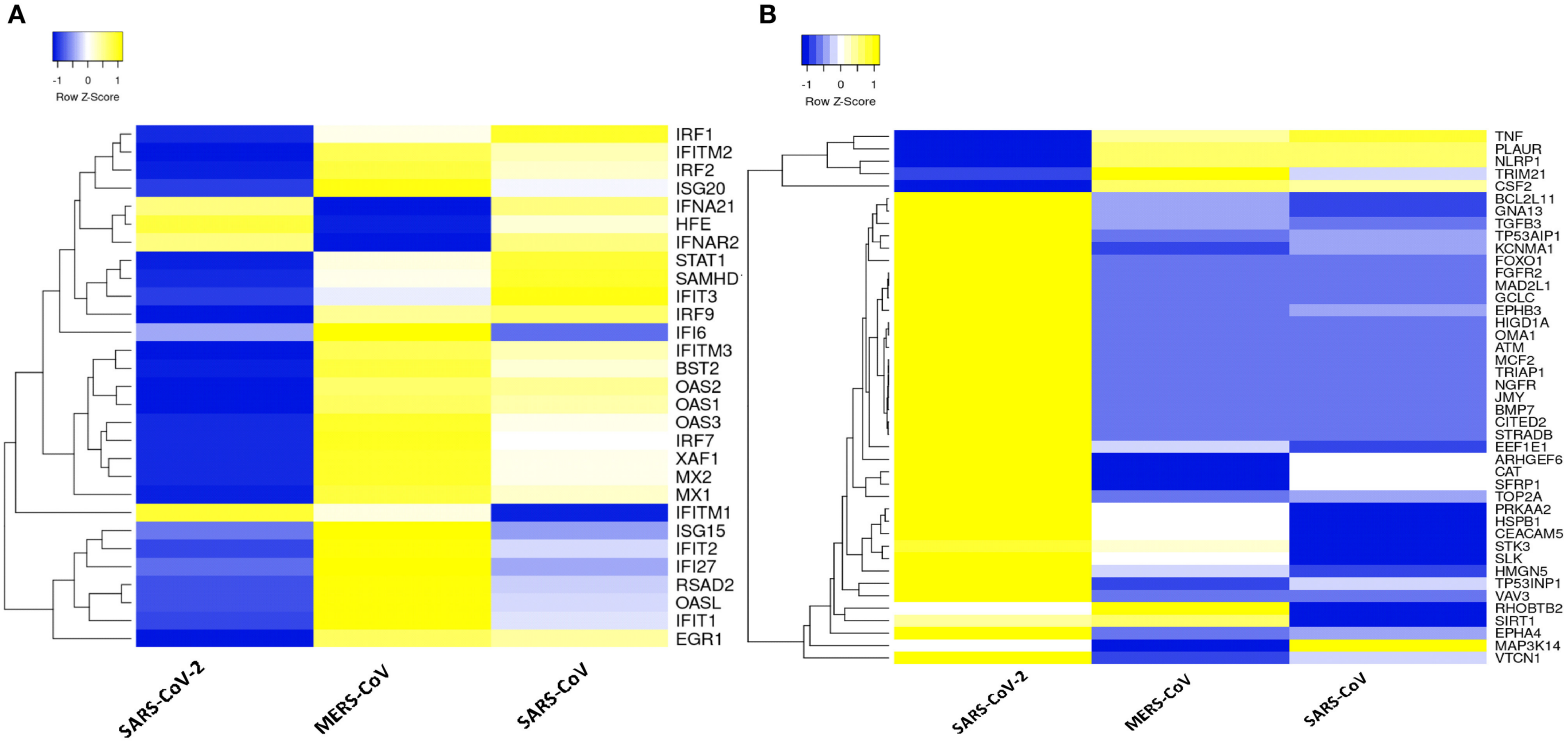
Type 1 IFN response and apoptotic genes signature in three coronaviruses. **(A)** Heatmap representation of expression profiles for the type 1 IFN response genes (GO:0060337) between coronaviruses. **(B)** Heatmap representation of expression profiles for the apoptotic gene signatures (GO:0042981) between coronaviruses. Clustering of selected genes on the heatmap was performed by hierarchical clustering algorithm using Euclidean distance measure. Expression scale: blue (low expression) to yellow (high expression).

Dissecting the DEGs involved in IFN response to coronavirus infection in primary human lung epithelial cells revealed that SARS-CoV-2 elicits a muted response that lacks robust induction of a subset of cytokines including the type I IFN compared to the response to MERS-CoV and SARS-CoV (Figure 3A). Furthermore, our analysis revealed that in desperate bid to control the viral propagation, SARS-CoV-2 infection induced several apoptosis-related genes in human lung epithelial cells compared to responses to MERS-CoV and SARS-CoV (Figure 3B).

### Downstream Analysis of DEGs Unique to SARS-CoV-2 and Network-Based Meta-Analysis Led to Pathways and Hub Genes Related to Inflammation and Vascular Dysfunction

**Figure 4:**
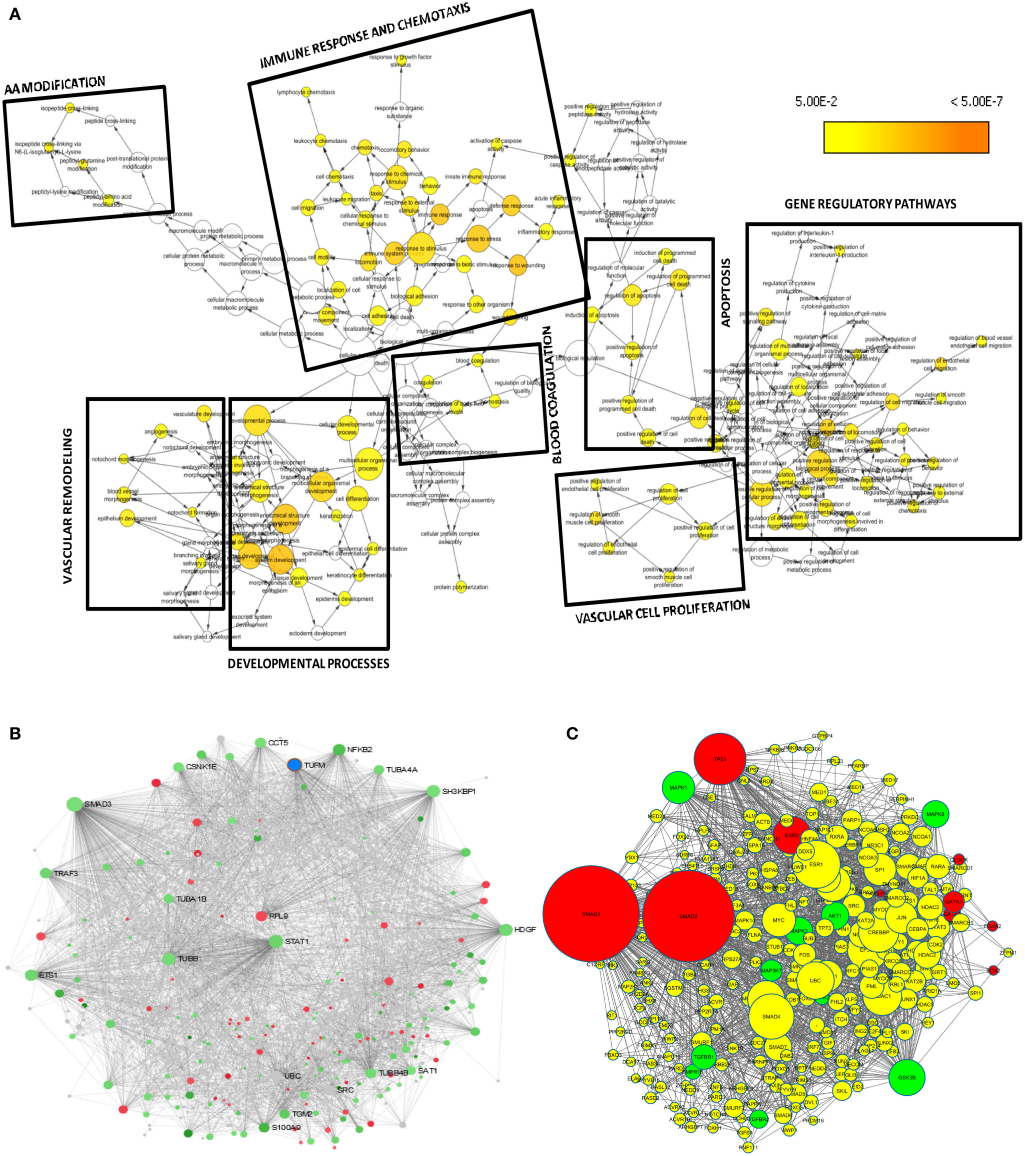
Downstream analysis of SARS-CoV-2–specific DEGs. **(A)** Overrepresentation of pathways and Gene Ontology categories in biological networks identified from DEGs unique to SARS-CoV-2. Significantly overrepresented biological processes based on GO terms were visualized in Cytoscape. The size of a node is proportional to the number of targets in the GO category. The color represents enrichment significance—the deeper the color on a color scale, the higher the enrichment significance. *p* values were adjusted using a Benjamini and Hochberg FDR correction. Analysis revealed the enriched pathways associated with immune responses and chemotaxis, blood coagulation, apoptosis signaling pathway, vasculature remodeling, and vascular cell proliferation. **(B)** Network-based analysis of hub DEGs. Interaction network of SARS-CoV-2 unique genes; red nodes represent increased and green nodes represent decreased DEGs. **(C)** Regulatory gene network analysis of the top 10 enriched kinases (green) and transcription factors (red) using SARS-CoV-2 specific DEGs. Yellow nodes represent the intermediate proteins in the regulatory network. Node size represents the significance of protein based on *p* value; the bigger the node size, the higher the significance value.

A set of 221 DEGs unique to SARS-CoV-2 underwent downstream analysis to understand the enriched pathways and associated hub genes. Using BinGO enrichment clusters of biological process (GO terms) associated with unique DEGs of SARS-CoV-2 was generated, which revealed enriched pathway clusters associated with immune responses/chemotaxis, blood coagulation, apoptosis, vascular remodeling, and vascular cell proliferation (Figure 4A). Supplementary Dataset 4 compiles the complete list of GO: terms enriched in DEGs associated with SARS-CoV-2 infection. We generated a PPI network by integrating the InnateDB interactome with the original seed of 221 DEGs. An expanded PPI network was generated with 2,542 nodes representing the proteins and 4,457 edges representing the interaction between these proteins. Network-based hub DEG analysis (Figure 4B) identified ribosomal protein L9 (*RPL9*) and SMAD family member 3 (*SMAD3*) to be the most highly ranked hub genes that increased and decreased among the DEGs, respectively, based on betweenness centrality and degree score. The list of top 15 hub genes based on network topology scores is shown in Table 4.

**Table 4:** Network-based hub genes of SARS-CoV-2 specific DEGs.

**Label**	**Degree**	**Betweenness**	**Expression**
SMAD3	330	705,502.9	−0.32876
STAT1	223	437,249.9	−0.4737
SH3KBP1	178	358,520.9	−0.32778
HDGF	177	330,006.3	−0.38192
TUBB	173	263,526.3	−0.34572
NFKB2	138	213,825.4	−0.86093
ETS1	133	255,847.5	−0.43368
TUFM	116	174,412.6	−0.27641
UBC	106	115,3510	0
TRAF3	104	1,793,40.7	−0.43528
CCT5	99	147,216.3	−0.31476
RPL9	98	114,815.3	0.38872
TUBB4B	86	79,259.32	−0.38926
CSNK1E	84	158,782.5	−0.3193
S100A9	83	123,102.9	−1.0222

*Top 15 genes prioritized based on topological parameters are shown. Expression levels are incorporated in the table from the transcriptome analysis result*.

### Identification of the Transcription Factors and Regulatory Kinases Network Upstream to the Unique DEGs Obtained From SARS-CoV-2

To understand what lies to the upstream of the unique DEGs identified from the SARS-CoV-2 infection, we used X2K bioinformatics tool. The regulatory gene network analysis resulted in identification of transcription factors and kinases related to the DEGs. Network in Figure 4C shows the top kinases and transcription factors related to our DEGs. The list of top 10 ranked transcription factors and protein kinases is shown in Supplementary Table 4. This analysis revealed the most important regulatory gene candidates that may be involved in the formation of regulatory complexes. Mitogen-activated protein kinase 1 (*MAPK1*) and MAPK3 are among the top kinases, whereas SMAD3 and SMAD2 are among the top transcription factors associated with the unique DEGs from lung epithelial cells infected with SARS-CoV-2.

### Distinct Pathways and Gene Signatures Associated With SARS-CoV-2

After we removed the DEGs shared by three coronaviruses (17 DEGs) and those shared between SARS-CoV-2 and MERS-CoV (91 DEGs); and SARS-CoV-2 and SARS-CoV (9 DEGs), a total of 221 DEGs remained that was specific to SARS-CoV-2 (Figure 2A and Supplementary Dataset 1). Our interest was to identify the distinct pathways that may participate in the pathogenesis of COVID-19. Using the list of SARS-CoV-2–specific DEGs, we conducted biological process (GO) analysis on the unique set of DEGs using a Cytoscape plugin, BinGO tool. Table 5 depicts the most distinct pathways and their associated representative genes with its known function and expression fold change that may have implication on the disease pathogenesis. In the analysis, the following pathways were enriched. In the apoptosis-related pathway, the expression of neuropilin 1 (*NRP1*), forkhead box O1 (*FOXO1*), and tumor protein p53 inducible nuclear protein 1 (*TP53INP1*) is increased, whereas that of colony-stimulating factor 2 (*CSF2*) and NLR family pyrin domain containing 1 (*NLRP1*) is decreased. Acute inflammation–related genes included IL-6 receptor (*IL-6R*) that increased and serpin family A member 3 (*SERPINA3*), complement component 1s (*C1S*), serum amyloid A2 (*SAA2*), and complement factor B (*CFB*) that decreased. Among vascular dysfunction–related genes, vav guanine nucleotide exchange factor 3 (*VAV3*) and transcription factor 4 (TCF4) are increased, while thymidine phosphorylase (*TYMP*) and nuclear receptor subfamily 2 group F member 2 (*NR2F2*) are decreased. Genes related to blood coagulation included coagulation factor III/tissue factor (*F3*) and protein S (*PROS1*) are increased, whereas IFN-γ receptor 1 (*IFNGR1*), integrin subunit β3 (*ITGB3*), and tissue factor pathway inhibitor 2 (*TFPI2*) are decreased. Several pathways associated with cardiovascular dysfunction were enriched by the unique set of DEGs specific to SARS-CoV-2 infection. Table 6 summarizes the pathways and their associated genes that might play a role in cardiovascular complications.

**Table 5:** Distinct pathways and related genes associated with SARS-CoV-2.

**Gene**	**Gene name**	**Role**	**logFold change**	**Adj p-val**
**Apoptosis-related genes in COVID-19–infected lung epithelial cells**				
NRP1	Neuropilin 1	Regulation of apoptotic pathways	0.332	0.023326
FOXO1	Forkhead box O1	Important regulator of cell death acting downstream of several signaling pathways including CDK1, PKB/AKT1 and STK4/MST1	0.432	0.014544
TP53INP1	Tumor Protein P53 inducible nuclear protein 1	Induce cell death by an autophagy and caspase-dependent mechanism	0.467	0.002053
CSF2	Colony-stimulating factor 2	Inhibits induction of apoptosis in several cell types	−1.136	2.92E-11
NLRP1	NLR family pyrin domain containing 1	Can induce pyroptosis, an inflammatory form of programmed cell death	−0.302	0.032517
**Acute inflammation–related genes in COVID-19–infected lung epithelial cells**				
SERPINA3	Serpin family A member 3	Is a typical acute-phase protein secreted into the circulation during acute and chronic inflammation	−1.194	1.26E-20
C1S	Complement C1s	Subunit of first component of the classical pathway of the complement system, released in acute inflammatory response	−0.525	0.01783
SAA2	Serum amyloid A2	SAA2 encode acute phase proteins (ASAA) that are released in response to inflammatory stimuli	−2.067	4.79E-77
IL6R	Interleukin 6 receptor	Regulation of the immune response, acute-phase reactions and hematopoiesis	0.498	0.000642
CFB	Complement factor B	Important component of complement system and inflammatory response	−1.563	5.47E-41
**Vascular dysfunction–related genes in COVID-19–infected lung epithelial cells**				
VAV3	Vav Guanine Nucleotide Exchange Factor 3	Vav3-induced cytoskeletal dynamics contribute to heterotypic properties of endothelial barriers, thus important in vascular stability	0.531	0.000426
TYMP	Thymidine phosphorylase	Role in maintaining the integrity of the blood vessels and angiogenesis	−0.755	6.23E-06
TCF4	Transcription factor 4	No known direct role	0.472	0.00286
NR2F2	Nuclear receptor subfamily 2 group F member 2	Suppression of COUP-TFII in venous ECs switched its phenotype toward proatherogenic by up-regulating the expression of inflammatory genes and down-regulating antithrombotic genes	−0.370	0.007664
**Blood coagulation–related genes in COVID-19–infected lung epithelial cells**				
F3	Coagulation factor III, tissue factor	Enables cells to initiate the blood coagulation cascades, and it functions as the high-affinity receptor for the coagulation factor VII	0.384	0.0002
PROS1	Protein S	Anticoagulant plasma protein, which helps to prevent coagulation and stimulating fibrinolysis	0.541	0.000358
IFNGR1	Interferon γ receptor 1	No known direct role	−0.365	0.01711
ITGB3	Integrin subunit beta 3	Rapid platelet aggregation, which physically plugs ruptured endothelial surface	−0.747	6.52E-05
TFPI2	Tissue factor pathway inhibitor 2	Protein can inhibit a variety of serine proteases including factor VIIa/tissue factor, thus suppress coagulation	−0.316	0.022707

*List of differentially expressed pathways and genes associated with them. Possible roles were extracted from STRING database, and the expression values were added from the RNA-seq analysis results*.

**Table 6:** Cardiovascular dysfunction–related pathways and genes in SARS-CoV-2 infection.

**Cardiovascular dysfunction–related pathway/terms**	**GO-ID**	***p* value**	**Adjusted *p* value**	**Overlap**	**Associated genes**
Vasculature development	1944	4.77E-05	3.35E-03	13/273	VAV3, NRP1, ITGB3, NR2F2, FOXO1, TYMP, ZC3H12A, COL8A1, TCF4, ITGA5, EPHA, TGM2, S100A7
Blood vessel morphogenesis	48514	1.20E-04	6.14E-03	11/220	VAV3, NRP1, ITGB3, ZC3H12A, COL8A1, NR2F2, ITGA5, EPHA2, TYMP, TGM2, S100A7
Leukocyte chemotaxis	30595	1.84E-04	7.54E-03	5/41	PDGFB, SAA2, IL6R, S100A9, CXCL16
Cell adhesion	7155	2.79E-04	9.71E-03	21/710	NRP1, DST, ITGB3, PCDH7, COL12A1,TNC, NPNT, NID1, F3, MTSS1, FLRT3, FEZ1, CDH10, LY6D, CLCA2, COL8A1, FAT2, NRCAM, FAT4, ITGA5, DSG3
Regulation of endothelial cell proliferation	1936	3.15E-04	1.01E-02	5/51	BMP2, ITGB3, PDGFB, NR2F2, F3
Regulation of chemotaxis	50920	4.63E-04	1.37E-02	5/51	SMAD3, PDGFB, F3, IL6R, S100A7
Positive regulation of cell death	10942	6.32E-04	1.69E-02	15/449	VAV3, NRP1, SMAD3, STAT1, TUBB, IGFBP3, ETS1, ALDH1A3, BMP2, TRAF3, KCNMA1, TP53INP1, NLRP1, BID, TGM2
Positive regulation of endothelial cell proliferation	1938	6.92E-04	1.80E-02	4/32	BMP2, ITGB3, PDGFB, F3
Angiogenesis	1525	7.31E-04	1.85E-02	8/152	VAV3, NRP1, ITGB3, ZC3H12A, COL8A1, ITGA5, TYMP, S100A7
Leukocyte migration	50900	1.14E-03	2.67E-02	5/62	PDGFB, SAA2, IL6R, S100A9, CXCL16
Regulation of smooth muscle cell migration	14910	1.97E-03	3.86E-02	3/20	IGFBP3, PDGFB, F3
Lymphocyte chemotaxis	48247	2.34E-03	4.23E-02	2/6	SAA2, CXCL16
Regulation of blood vessel endothelial cell migration	43535	2.62E-03	4.50E-02	3/22	EFNA1, PDGFB, EPHA2

*The corresponding p values are adjusted, based on the false discovery rate using the Benjamini–Hochberg procedure*.

## Discussion

In the present study, we focus on defining transcriptional responses to SARS-CoV-2 relative to MERS-CoV and SARS-CoV. A major goal was to discover unique cellular responses to SARS-CoV-2 among these three coronaviruses. We specifically selected datasets generated in cultured human lung epithelial cells infected with each of these three coronaviruses as this cell type is the major interface between the environment and the host and defends the lung against foreign substances and pathogens. In general, our data show that overall transcriptional footprints to SARS-CoV-2 infection were distinct from those to the other two coronaviruses. Despite the decreased expression of acute inflammatory and type I IFN genes in response to SARS-CoV-2, we observed increased expression of several genes associated with interleukin signaling, complement pathways, and chemokines. This finding echoes with the previously published study, which conducted RNAseq analysis to understand host transcriptional response to influenza A virus and SARS-CoV-2 in primary human bronchial epithelial cells (11). We used a publicly available subset of RNAseq data (GSE147507) from this study to compare it with independent datasets for SARS-CoV and MERS-CoV. It is worth mentioning that the list of DEGs unique to SARS-CoV-2 infection generated in our analysis was associated with coagulation and vascular function, which may explain why COVID-19 causes more systemic cardiovascular complications than do MERS and SARS (4, 8).

By analyzing RNAseq dataset of lung epithelial cells infected with SARS-CoV-2, we defined transcriptional signatures of 338 DEGs, including 92 increased and 246 decreased genes across the datasets. Among the top 10 increased DEGs, *IFITM10, CXCL14*, and *MYLK* are the most significantly increased genes. CXCL14 is a cytokine involved in immunoregulatory and inflammatory processes by mediating the chemotactic activity for monocytes and therefore can be implicated in the immune cell infiltration in the lung during SARS-CoV-2 infection (24). IFITM proteins family inhibit the entry of a large number of viruses; however, the exact role of IFITM10 as an antiviral agent remains unknown (25). SAA2 is the most significantly decreased gene in our analysis. SAA2 is a useful inflammatory marker in acute viral infections such as influenza, but its decreased expression in our analysis is consistent with the aberrant inflammatory response of SARS-CoV-2 infection (26). Viral infection is marked by the activation of immune system, which is evident from the enrichment of several pathways, including IL-17, TNF, and apoptosis signaling pathways among others (27). Patients who are infected with COVID-19 may develop pneumonia and progress to severe respiratory failure termed acute respiratory distress syndrome, which may result in the development of lung fibrosis (28). Consistent with this report, several genes such as colony-stimulating factor 3 (*CSF3*), endothelin 1 (*EDN1*), plasminogen activator, urokinase (*PLAU*), and *MMP9* were reported to be differentially expressed in SARS-CoV-2 infection.

Previous studies revealed that lung epithelial cells, macrophages, and dendritic cells express cytokines to some extent during major viral infection causing cytokine storm. Evidence for molecular mechanisms of cytokine storm in COVID-19 remains limited. Earlier studies have shown IFN-γ-related cytokine storm in SARS patients (29), while delayed induction of proinflammatory cytokines and suppression of innate antiviral response by the MERS-CoV (30). It is crucial to identify the primary source of the cytokine storm in response to SARS-CoV-2 infection and the virological mechanisms behind the cytokine storm. Consistent to this model, our analysis of shared DEGs between three coronaviruses resulted in 17 DEGs, among which most molecules are related to acute inflammation. It is evident that important acute inflammatory genes (e.g., *TNFAIP3, CXCL1*, and *TNF*) are decreased in SARS-CoV-2–infected cells compared to other coronaviruses. These results may suggest that the major source of cytokine storm in COVID-19 is not lung epithelial cells, but possibly immune cell types. These aberrant transcriptional responses to SARS-CoV-2 may indicate low responses to infection, resulting in prolonged viral replication and serious lung damage in COVID-19 (31).

Our study linked DEGs unique to SARS-CoV-2 infection with pathway clusters related to immune responses, blood coagulation, apoptosis, and vascular remodeling. Apoptosis, which is a defense mechanism of hosts against the viral infection, depends on the rapid programmed cell death to curtail viral spread. Previous studies reported that SARS-CoV has evolved sophisticated molecular strategies to trigger host cell apoptotic defenses (32, 33). In our data, SARS-CoV-2 infection increased the expression of *NRP1* and *FOXO1*. NRP1 has a regulatory role of apoptotic pathways, whereas FOXO1 is an important regulator of cell death acting downstream of several signaling pathways, including CDK1, PKB/AKT1, and STK4/MST1 (34).

Evidence suggests elevation of d-dimer and fibrin/fibrinogen degradation products in patients with COVID-19, highlighting aggravated blood coagulation (8). A recent clinical study examined seven lungs obtained during autopsy from patients with COVID-19 (35). The study observed vascular endothelial injury and widespread thrombus formation in pulmonary vessels. Immunohistochemical staining of pulmonary vasculature of COVID-19 showed alveolar capillary microthrombi were nine times as prevalent in patients with COVID-19 compared with influenza patients. In consistent with these reports, we identified coagulation-related genes in SARS-CoV-2 infection, including tissue factor that initiates the external coagulation cascades. In contrast, SARS-CoV-2 suppressed the expression of antithrombotic gene TFPI2, which inhibits a variety of serine proteases including factor VIIa/tissue factor complex.

Despite the clinical impact, the information on the mechanisms of COVID-19 and its cardiovascular complications remains limited. A systems approach, involving unbiased bioinformatics and network analysis, may help to identify causative genes and integrated pathways as drug targets for the improvement in disease management (36). Network-based analysis of hub genes in the DEGs dataset unique to SARS-CoV-2 infection resulted in prioritization of RPL9 as the most highly ranked DEG that had increased expression, based on betweenness centrality and degree score. The increased expression of RPL9, a ribosomal protein, can be attributed to the fact that virus hijacks the translational machinery of the host for its survival by the mechanisms such as ribosome shunting and phosphorylation of ribosomal proteins (37, 38).

Regulatory gene network analysis helps to understand what lies upstream of the DEGs in cells infected with SARS-CoV-2. It is important to find out the regulatory kinases and transcription factors as they participate in the pathogenesis and the progression of the virus infection (39). Among several kinases regulating the expression of DEGs expression in our analysis, genes involved in MAPK cascades (*MAPK1, MAPK2, MAPK8*, and *MAP3K7*) have roles in host response to viral infection (40). SMAD3, an effector molecule in the transforming growth factor-β signaling pathway, is also an interesting candidate in our analysis as it was the top hub gene in our network analysis and also the most significant transcription factor in our regulatory gene analysis. In the present study, SARS-CoV-2 reduced SMAD3 expression, which is consistent with previous findings on the decreased expression of SMAD3 during viral infection to overtake the host innate antiviral mechanism (41, 42).

In conclusion, our study provides the snapshot of transcriptional host responses to SARS-CoV-2 infection, in which expression of various inflammatory genes is decreased. These results may explain why many infected individuals show no symptoms. Furthermore, our study revealed that SARS-CoV-2 elicits muted antiviral type I IFN response, which may result in prolonged viral replication. These findings may also explain why SARS-CoV-2 infection has a longer incubation period than other coronavirus infections. Our analysis revealed expression of several genes related to apoptosis, coagulation, and vascular function, which may contribute to cardiovascular complications. Furthermore, our study has identified a novel set of candidate transcriptomic signatures unique to SARS-CoV-2 infection, which may guide the initial efforts in the development of diagnostic or therapeutic tools for COVID-19.

## Data Availability Statement

The original contributions presented in the study are included in the article/Supplementary Material, further inquiries can be directed to the corresponding author/s.

## Author Contributions

PJ, AV, and MA conceived, coordinated, and designed the study. PJ and AV retrieved the datasets, did the analysis, and wrote the manuscript. MA did the editing and result interpretation. AH and SU edited and provided technical advices on the study. All authors contributed to the article and approved the submitted version.

## Conflict of Interest

The authors declare that the research was conducted in the absence of any commercial or financial relationships that could be construed as a potential conflict of interest.

## References

[1] Thao TTN Labroussaa F Ebert N V'kovski P Stalder H Portmann J. Rapid reconstruction of SARS-CoV-2 using a synthetic genomics platform. Nature. (2020) 582:561–5. 10.1038/s41586-020-2294-9 32365353

[2] Zhou P Yang X-L Wang X-G Hu B Zhang L Zhang W. A pneumonia outbreak associated with a new coronavirus of probable bat origin. Nature. (2020) 579:270–3. 10.1038/s41586-020-2951-z 32015507 PMC7095418

[3] Madjid M Safavi-Naeini P Solomon SD Vardeny O. Potential effects of coronaviruses on the cardiovascular system: a review. JAMA Cardiol. (2020) 5:831–40. 10.1001/jamacardio.2020.1286 32219363

[4] Clerkin KJ Fried JA Raikhelkar J Sayer G Griffin JM Masoumi A COVID-19 and cardiovascular disease. Circulation. (2020) 141:1648–55. 10.1161/CIRCULATIONAHA.120.046941 32200663

[5] Zou L Ruan F Huang M Liang L Huang H Hong Z. SARS-CoV-2 viral load in upper respiratory specimens of infected patients. N Engl J Med. (2020) 382:1177–9. 10.1056/NEJMc2001737 32074444 PMC7121626

[6] Peiris JS Chu CM Cheng VC Chan KS Hung IF Poon LL. Clinical progression and viral load in a community outbreak of coronavirus-associated SARS pneumonia: a prospective study. Lancet. (2003) 361:1767–72. 10.1016/S0140-6736(03)13412-5 12781535 PMC7112410

[7] Oh M-d Park WB Choe PG Choi S-J Kim J-I Chae J. Viral load kinetics of MERS coronavirus infection. N Engl J Med. (2016) 375:1303–5. 10.1056/NEJMc1511695 27682053

[8] Connors JM Levy JH. COVID-19 and its implications for thrombosis and anticoagulation. Blood. (2020) 135:2033–40. 10.1182/blood.2020006000 32339221 PMC7273827

[9] Middeldorp S Coppens M van Haaps TF Foppen M Vlaar AP Müller MCA Incidence of venous thromboembolism in hospitalized patients with COVID-19. J Thromb Haemost. (2020) 18:1995–2002. 10.20944/preprints202004.0345.v1 32369666 PMC7497052

[10] Tang N Bai H Chen X Gong J Li D Sun Z Anticoagulant treatment is associated with decreased mortality in severe coronavirus disease 2019 patients with coagulopathy. J Thromb Haemost. (2020) 18:1094–9. 10.1111/jth.14817 32220112 PMC9906401

[11] Blanco-Melo D Nilsson-Payant BE Liu WC Uhl S Hoagland D Moller R. Imbalanced host response to SARS-CoV-2 drives development of COVID-19. Cell. (2020) 181:1036–45.e9. 10.1016/j.cell.2020.04.026 32416070 PMC7227586

[12] Love MI Huber W Anders S. Moderated estimation of fold change and dispersion for RNA-seq data with DESeq2. Genome Biol. (2014) 15:550. 10.1186/s13059-014-0550-8 25516281 PMC4302049

[13] Xia J Benner MJ Hancock RE. NetworkAnalyst–integrative approaches for protein-protein interaction network analysis and visual exploration. Nucleic Acids Res. (2014) 42:W167–74. 10.1093/nar/gku443 24861621 PMC4086107

[14] Lin SM Du P Huber W Kibbe WA. Model-based variance-stabilizing transformation for Illumina microarray data. Nucleic Acids Res. (2008) 36:e11. 10.1093/nar/gkm1075 18178591 PMC2241869

[15] Yoshikawa T Hill TE Yoshikawa N Popov VL Galindo CL Garner HR. Dynamic innate immune responses of human bronchial epithelial cells to severe acute respiratory syndrome-associated coronavirus infection. PLoS ONE. (2010) 5:e8729. 10.1371/journal.pone.0008729 20090954 PMC2806919

[16] Ritchie ME Phipson B Wu D Hu Y Law CW Shi W. limma powers differential expression analyses for RNA-sequencing and microarray studies. Nucleic Acids Res. (2015) 43:e47. 10.1093/nar/gkv007 25605792 PMC4402510

[17] Witten DM Tibshirani R Hastie T. A penalized matrix decomposition, with applications to sparse principal components and canonical correlation analysis. Biostatistics. (2009) 10:515–34. 10.1093/biostatistics/kxp008 19377034 PMC2697346

[18] Chen EY Tan CM Kou Y Duan Q Wang Z Meirelles GV. Enrichr: interactive and collaborative HTML5 gene list enrichment analysis tool. BMC Bioinform. (2013) 14:128. 10.1186/1471-2105-14-128 23586463 PMC3637064

[19] Ashburner M Ball CA Blake JA Botstein D Butler H Cherry JM. Gene ontology: tool for the unification of biology. The Gene Ontology Consortium. Nat Genet. (2000) 25:25–9. 10.1038/75556 10802651 PMC3037419

[20] Shannon P Markiel A Ozier O Baliga NS Wang JT Ramage D. Cytoscape: a software environment for integrated models of biomolecular interaction networks. Genome Res. (2003) 13:2498–504. 10.1101/gr.1239303 14597658 PMC403769

[21] Maere S Heymans K Kuiper M. BiNGO: a Cytoscape plugin to assess overrepresentation of gene ontology categories in biological networks. Bioinformatics. (2005) 21:3448–9. 10.1093/bioinformatics/bti551 15972284

[22] Lachmann A Xu H Krishnan J Berger SI Mazloom AR Ma'ayan A. ChEA: transcription factor regulation inferred from integrating genome-wide ChIP-X experiments. Bioinformatics. (2010) 26:2438–44. 10.1093/bioinformatics/btq466 20709693 PMC2944209

[23] Chen EY Xu H Gordonov S Lim MP Perkins MH Ma'ayan A. Expression2Kinases: mRNA profiling linked to multiple upstream regulatory layers. Bioinformatics. (2012) 28:105–11. 10.1093/bioinformatics/btr625 22080467 PMC3244772

[24] Schett G Sticherling M Neurath MF. COVID-19: risk for cytokine targeting in chronic inflammatory diseases? Nat Rev Immunol. (2020) 20:271–2. 10.1038/s41577-020-0312-7 32296135 PMC7186927

[25] Qian J Le Duff Y Wang Y Pan Q Ding S Zheng YM. Primate lentiviruses are differentially inhibited by interferon-induced transmembrane proteins. Virology. (2015) 474:10–8. 10.1016/j.virol.2014.10.015 25463599 PMC4581848

[26] Vollmer AH Gebre MS Barnard DL. Serum amyloid A (SAA) is an early biomarker of influenza virus disease in BALB/c, C57BL/2, Swiss-Webster, and DBA.2 mice. Antiviral Res. (2016) 133:196–207. 10.1016/j.antiviral.2016.08.011 27523492 PMC5042138

[27] Mukherjee S Lindell DM Berlin AA Morris SB Shanley TP Hershenson MB. IL-17-induced pulmonary pathogenesis during respiratory viral infection and exacerbation of allergic disease. Am J Pathol. (2011) 179:248–58. 10.1016/j.ajpath.2011.03.003 21703407 PMC3123803

[28] George PM Wells AU Jenkins RG. Pulmonary fibrosis and COVID-19: the potential role for antifibrotic therapy. Lancet Respir Med. (2020) 8:807–15. 10.1016/S2213-2600(20)30225-3 32422178 PMC7228727

[29] Huang KJ Su IJ Theron M Wu YC Lai SK Liu CC An interferon-gamma-related cytokine storm in SARS patients. J Med Virol. (2005) 75:185–94. 10.1002/jmv.20255 15602737 PMC7166886

[30] Lau SKP Lau CCY Chan KH Li CPY Chen H Jin DY. Delayed induction of proinflammatory cytokines and suppression of innate antiviral response by the novel Middle East respiratory syndrome coronavirus: implications for pathogenesis and treatment. J Gen Virol. (2013) 94 (Pt 12):2679–90. 10.1099/vir.0.055533-0 24077366

[31] Blanco-Melo D Nilsson-Payant BE Liu W-C Møller R Panis M Sachs D SARS-CoV-2 launches a unique transcriptional signature from *in vitro, ex vivo*, and *in vivo* systems. bioRxiv. (2020). 10.1101/2020.03.24.004655

[32] Krahling V Stein DA Spiegel M Weber F Muhlberger E. Severe acute respiratory syndrome coronavirus triggers apoptosis via protein kinase R but is resistant to its antiviral activity. J Virol. (2009) 83:2298–309. 10.1128/JVI.01245-08 19109397 PMC2643707

[33] Tan YX Tan TH Lee MJ Tham PY Gunalan V Druce J. Induction of apoptosis by the severe acute respiratory syndrome coronavirus 7a protein is dependent on its interaction with the Bcl-XL protein. J Virol. (2007) 81:6346–55. 10.1128/JVI.00090-07 17428862 PMC1900074

[34] Fu Z Tindall DJ. FOXOs, cancer and regulation of apoptosis. Oncogene. (2008) 27:2312–9. 10.1038/onc.2008.24 18391973 PMC2819403

[35] Ackermann M Verleden SE Kuehnel M Haverich A Welte T Laenger F. Pulmonary vascular endothelialitis, thrombosis, and angiogenesis in Covid-19. N Engl J Med. (2020) 383:120–8. 10.1056/NEJMoa2015432 32437596 PMC7412750

[36] Barabasi AL Gulbahce N Loscalzo J. Network medicine: a network-based approach to human disease. Nat Rev Genet. (2011) 12:56–68. 10.1038/nrg2918 21164525 PMC3140052

[37] Hertz MI Landry DM Willis AE Luo G Thompson SR. Ribosomal protein S25 dependency reveals a common mechanism for diverse internal ribosome entry sites and ribosome shunting. Mol Cell Biol. (2013) 33:1016–26. 10.1128/MCB.00879-12 23275440 PMC3623076

[38] Meyuhas O. Physiological roles of ribosomal protein S6: one of its kind. Int Rev Cell Mol Biol. (2008) 268:1–37. 10.1016/S1937-6448(08)00801-0 18703402

[39] Ramezani A Nahad MP Faghihloo E. The role of Nrf2 transcription factor in viral infection. J Cell Biochem. (2018) 119:6366–82. 10.1002/jcb.26897 29737559

[40] Chu WM Ostertag D Li ZW Chang L Chen Y Hu Y. JNK2 and IKKbeta are required for activating the innate response to viral infection. Immunity. (1999) 11:721–31. 10.1016/S1074-7613(00)80146-6 10626894

[41] Gough NR Enhancing and inhibiting TGF-β signaling in infection. Sci Signal. (2015) 8:ec9-ec. 10.1126/scisignal.aaa6549

[42] Nie Y Cui D Pan Z Deng J Huang Q Wu K. HSV-1 infection suppresses TGF-beta1 and SMAD3 expression in human corneal epithelial cells. Mol Vis. (2008) 14:1631–8. 18776948 PMC2529468

