# Supplementary material for: Gene Expression Profiling Reveals the Shared and Distinct Transcriptional Signatures in Human Lung Epithelial Cells Infected With SARS-CoV-2, MERS-CoV, or SARS-CoV: Potential Implications in Cardiovascular Complications of COVID-19
Source: Front Cardiovasc Med. 2021 Jan 15;7:623012. doi: 10.3389/fcvm.2020.623012 (PMC7844200; doi:10.3389/fcvm.2020.623012)
Supplement: Supplementary file 1 [file Table_1.DOC]

**SUPPLEMENTARY INFORMATION**

**Gene expression profiling reveals the shared and distinct transcriptional signatures in human lung epithelial cells infected with SARS-CoV-2, MERS-CoV or SARS-CoV: Potential implications in cardiovascular complications of COVID-19**

Prabhash Kumar Jha^1#^, Aatira Vijay^4#^, Arda Halu^2,3^, Shizuka Uchida^5^ and Masanori Aikawa^1,2,3 *^

^1^Center for Excellence in Vascular Biology, Brigham and Women's Hospital, Harvard Medical School, Boston, USA

^2^Center for Interdisciplinary Cardiovascular Sciences, Brigham and Women's Hospital, Harvard Medical School, Boston, USA

^3^Channing Division of Network Medicine, Brigham and Women's Hospital, Harvard Medical School, Boston, USA

^4^Department of Cardiovascular and Metabolic Sciences, Lerner Research Institute, Cleveland Clinic, Cleveland, OH, USA

^5^ Center for RNA Medicine, Department of Clinical Medicine, Aalborg University, Copenhagen, Denmark

#These authors contributed equally

**^*^Correspondence:**

Masanori Aikawa, MD, PhD;

Center for Excellence in Vascular Biology, Brigham and Women's Hospital, HarvardMedical School, 77 Ave Louis Pasteur, NRB741, Boston, USA 02115; [maikawa@bwh.harvard.edu](mailto:maikawa@bwh.harvard.edu)

**Supplementary datasheet 1**- List of differentially expressed genes in human lung epithelial cell infected with SARS-CoV-2; sheet 1- all significant DEGs and sheet 2- DEGs unique to SARS-CoV-2.

**Supplementary datasheet 2**- List of differentially expressed genes in human lung epithelial cell infected with MERS-CoV; sheet 1- all significant DEGs and sheet 2- DEGs unique to MERS-CoV.

**Supplementary datasheet 3**- List of differentially expressed genes in human lung epithelial cell infected with SARS-CoV; sheet 1- all significant DEGs and sheet 2- DEGs unique to SARS-CoV.

**Supplementary datasheet 4**- List of gene ontology pathways associated with unique DEGs of human lung epithelial cell infected with SARS-CoV-2.

**Figure S1-** Illustration of QC plots for SARS-CoV-2 dataset, before and after normalization: (A) Box plot (B) Density plot and (C) PCA plot. Green- Control sample and Red- Infected sample

**Figure S2-** Illustration of QC plots for MERS-CoV dataset, before and after normalization: (A) Box plot (B) Density plot and (C) PCA plot. Green- Control sample and Red- Infected sample

**Figure S3-** Illustration of QC plots for SARS-CoV dataset, before and after normalization: (A) Box plot (B) Density plot and (C) PCA plot. Green- Control sample and Red- Infected sample


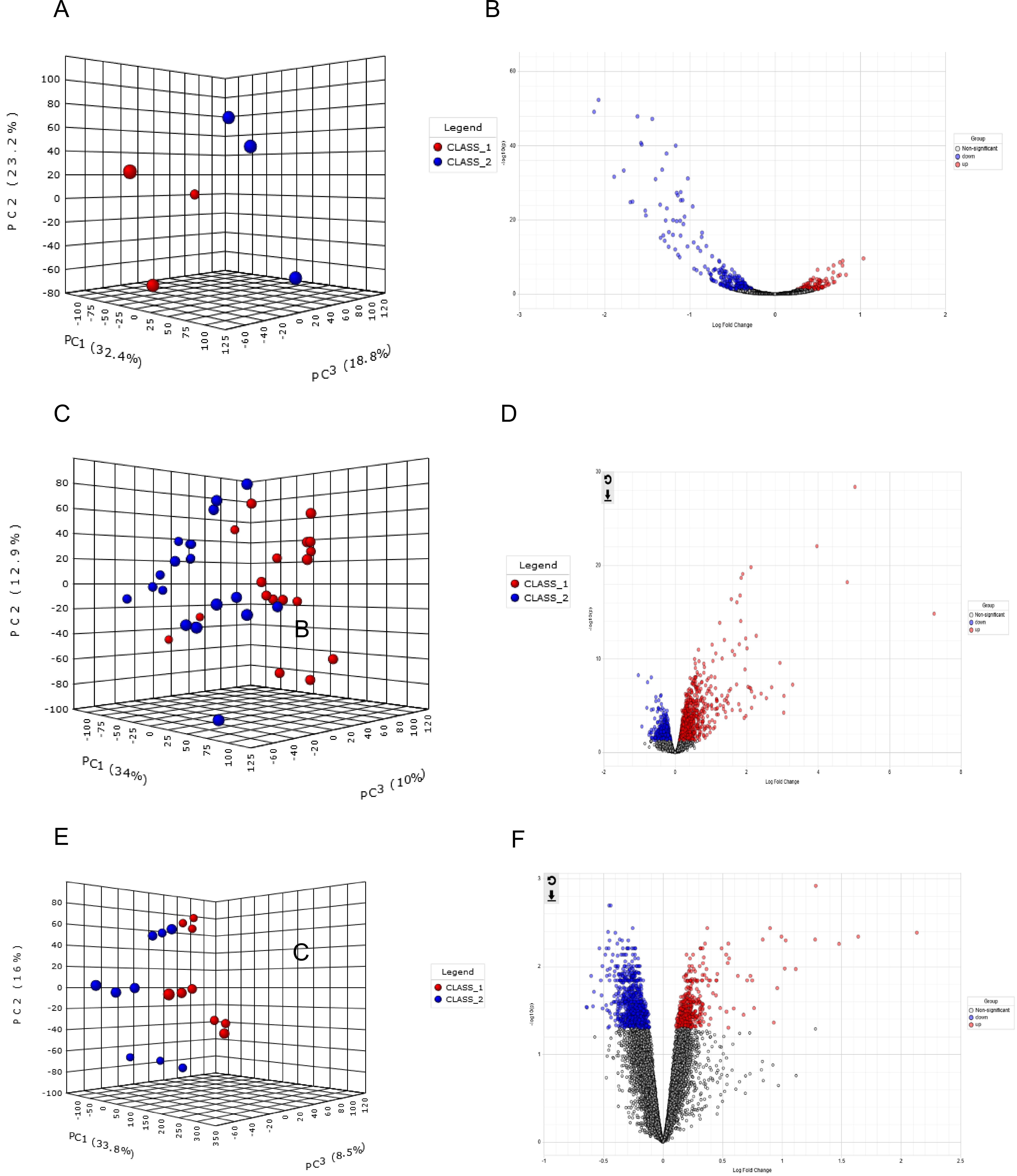
**Figure S4**- SARS-CoV-2- (A) Principal component analysis of sample clusters. Red- Control, Blue- Infected samples. (B) Volcano plot of DEGs. Blue- decreased DEGs, Red- increased DEGs and Grey- Non-significant DEGs. MERS-CoV- (C) Principal component analysis of sample clusters. Red- Control, Blue- Infected samples. (D) Volcano plot of DEGs. Blue- decreased DEGs, Red- increased DEGs and Grey- Non-significant DEGs and SARS-CoV- (E) Principal component analysis of sample clusters. Red- Control, Blue- Infected samples. (F) Volcano plot of DEGs. Blue- decreased DEGs, Red- increased DEGs and Grey- Non-significant DEGs.

| **S. No.** | **GEO accession no.** | **Sample source** | **Samples( Ctl/inf)** | **Organism** | **Platform** | **Model of generating expression summaries** | **Reference** | |
| --- | --- | --- | --- | --- | --- | --- | --- | --- |
| ***SARS-CoV-2 DATASET*** | | | | | | | | |
| 1 | [GSE147507] | Human lung epithelial cells- primary human lung epithelium (NHBE) | (n=06) 03/03 | Homo Sapiens | Illumina NextSeq 500 | log2 transformed and  quantile normalized | | (12) |
| ***MERS-CoV DATASET*** | | | | | | | | |
| 2 | [GSE81909] | Human lung epithelial cells- Human airway epithelial cells | (n=40) 20/20 | Homo Sapiens | Agilent-014850 Whole Human Genome Microarray 4x44K G4112F | VSN and  quantile normalized | | (-) |
| ***SARS-CoV DATASET*** | | | | | | | | |
| 3 | [GSE17400] | Human lung epithelial cells- Human bronchial epithelial cells | (n=18) 09/09 | Homo Sapiens | [HG-U133_Plus_2] Affymetrix Human Genome U133 Plus 2.0 Array | VSN and  quantile normalized | | (16) |

**Table S1. Characteristics of individual studies included in the gene expression analysis.** GEO: Gene Expression Omnibus

| **GENE ID** | **EntrezID** | **Gene Name** | **AveExpr** | **P.Value** | **adj.P.Val** | **logFC** |
| --- | --- | --- | --- | --- | --- | --- |
| **DEGs with increased expression** | | | | | | |
| **SSX2** | 6757 | SSX family member 2 | 12.114 | 1.26E-18 | 1.50E-15 | 7.2468 |
| **FOS** | 2353 | "Fos proto-oncogene, AP-1 transcription factor subunit" | 13.371 | 3.62E-33 | 4.30E-29 | 5.0318 |
| **EGR2** | 1959 | early growth response 2 | 10.461 | 3.22E-22 | 6.38E-19 | 4.8148 |
| **FOSB** | 2354 | "FosB proto-oncogene, AP-1 transcription factor subunit" | 9.5203 | 1.53E-26 | 9.06E-23 | 3.9658 |
| **TSPAN18** | 90139 | tetraspanin 18 | 8.4769 | 2.87E-10 | 5.59E-08 | 3.2877 |
| **HSPA6** | 3310 | heat shock protein family A (Hsp70) member 6 | 9.6403 | 8.04E-09 | 8.92E-07 | 3.0445 |
| **CXCL10** | 3627 | C-X-C motif chemokine ligand 10 | 8.7434 | 1.18E-06 | 5.55E-05 | 3.0381 |
| **EGR3** | 1960 | early growth response 3 | 8.5618 | 6.08E-13 | 2.68E-10 | 2.9321 |
| **RSAD2** | 91543 | radical S-adenosyl methionine domain containing 2 | 10.647 | 6.19E-10 | 1.06E-07 | 2.6994 |
| **OASL** | 8638 | 2'-5'-oligoadenylate synthetase like | 10.185 | 1.53E-08 | 1.54E-06 | 2.5518 |
| **DEGs with decreased expression** | | | | | | |
| **TCERG1** | 10915 | transcription elongation regulator 1 | 12.105 | 1.65E-11 | 5.46E-09 | -1.0296 |
| **GPR55** | 9290 | G protein-coupled receptor 55 | 8.0925 | 7.43E-05 | 0.001645 | -0.93123 |
| **CSN3** | 1448 | casein kappa | 8.668 | 0.026852 | 0.11058 | -0.84779 |
| **SLC51A** | 200931 | solute carrier family 51 subunit alpha | 11.643 | 2.62E-07 | 1.65E-05 | -0.79217 |
| **NDUFAF4** | 29078 | NADH:ubiquinone oxidoreductase complex assembly factor 4 | 11.366 | 1.39E-10 | 3.11E-08 | -0.77305 |
| **KRTAP4-8** | 728224 | keratin associated protein 4-8 | 8.6199 | 0.001108 | 0.012476 | -0.73408 |
| **TAFA4** | 151647 | TAFA chemokine like family member 4 | 8.137 | 0.10943 | 0.26769 | -0.72385 |
| **HDHD5-AS1** | 1E+08 | HDHD5 antisense RNA 1 | 13.338 | 0.000474 | 0.0066 | -0.71489 |
| **RASGRP4** | 115727 | RAS guanyl releasing protein 4 | 12.779 | 0.011133 | 0.061531 | -0.68809 |
| **MYOZ2** | 51778 | myozenin 2 | 7.7801 | 0.00062 | 0.008088 | -0.67983 |

**Table S2. Top 20 DEGs identified in the MERS-CoV analysis.** Genes were ranked based on the log fold change and adjusted p-value (<0.05). The corresponding *p-value*s are adjusted, based on the false discovery rate using the Benjamini–Hochberg procedure.

| **Gene ID** | **EntrezID** | **Gene Name** | **AveExpr** | **P.Value** | **adj.P.Val** | **logFC** |
| --- | --- | --- | --- | --- | --- | --- |
| **DEGs with increased expression** | | | | | | |
| **PTX3** | 5806 | pentraxin 3 | 5.9749 | 2.07E-06 | 0.004115 | 2.1328 |
| **EGR1** | 1958 | early growth response 1 | 10.065 | 3.12E-06 | 0.004563 | 1.6399 |
| **EGR2** | 1959 | early growth response 2 | 6.4834 | 7.65E-06 | 0.005522 | 1.4789 |
| **JUN** | 3725 | "Jun proto-oncogene, AP-1 transcription factor subunit" | 9.3142 | 7.59E-08 | 0.001205 | 1.2836 |
| **IFNB1** | 3456 | interferon beta 1 | 7.4624 | 0.005129 | 0.051323 | 1.2811 |
| **HSPA6** | 3310 | heat shock protein family A (Hsp70) member 6 | 7.9174 | 4.08E-06 | 0.004929 | 1.2783 |
| **CXCL11** | 6373 | C-X-C motif chemokine ligand 11 | 7.1231 | 0.056831 | 0.17472 | 1.1191 |
| **FOS** | 2353 | "Fos proto-oncogene, AP-1 transcription factor subunit" | 9.1993 | 5.79E-05 | 0.010685 | 1.116 |
| **CCL20** | 6364 | C-C motif chemokine ligand 20 | 8.7075 | 5.72E-06 | 0.005047 | 1.0307 |
| **TNFAIP3** | 7128 | TNF alpha induced protein 3 | 8.9796 | 5.38E-05 | 0.010541 | 1.0224 |
| **DEGs with decreased expression** | | | | | | |
| **AKR1B10** | 57016 | aldo-keto reductase family 1 member B10 | 10.967 | 0.001049 | 0.028889 | -0.64352 |
| **IFI6** | 2537 | interferon alpha inducible protein 6 | 10.658 | 0.001088 | 0.029208 | -0.63885 |
| **MMP1** | 4312 | matrix metallopeptidase 1 | 10.644 | 0.000105 | 0.0128 | -0.60381 |
| **TMEM159** | 57146 | transmembrane protein 159 | 9.0156 | 0.00098 | 0.028384 | -0.58805 |
| **HCG11** | 493812 | HLA complex group 11 | 6.6951 | 0.000332 | 0.019398 | -0.57992 |
| **CYS1** | 192668 | cystin 1 | 7.9549 | 0.008163 | 0.0635 | -0.57306 |
| **LONRF2** | 164832 | LON peptidase N-terminal domain and ring finger 2 | 7.4033 | 0.000171 | 0.014385 | -0.52171 |
| **SLC16A7** | 9194 | solute carrier family 16 member 7 | 8.7399 | 0.000413 | 0.021275 | -0.51616 |
| **C3orf33** | 285315 | chromosome 3 open reading frame 33 | 5.5389 | 4.16E-05 | 0.009892 | -0.50896 |
| **PRDX5** | 25824 | peroxiredoxin 5 | 11.666 | 0.001184 | 0.03002 | -0.50764 |

**Table S3. Top 20 DEGs identified in the SARS-CoV analysis.** Genes were ranked based on the log fold change and adjusted p-value (<0.05). The corresponding *p-value*s are adjusted, based on the false discovery rate using the Benjamini–Hochberg procedure.

| **Enrichment Term** | **Pathway/Term ID** | **Overlap** | **GSEA library** | **Adjusted**  **P-value** |
| --- | --- | --- | --- | --- |
| **Oxidative Stress Induced Gene Expression Via Nrf2** | h arenrf2Pathway | 07/18 | Biocarta | 0.059478 |
| **Influenza A** | hsa05164 | 36/171 | KEGG | 3.14E-04 |
| **Apoptosis signaling pathway** | P00006 | 26/102 | Panther | 4.04E-04 |
| **Cytokine Signaling in Immune system** | R-HSA-1280215 | 122/620 | Reactome | 3.67E-11 |
| **Photodynamic therapy-induced NF-kB survival signaling** | WP3617 | 16/35 | Wikipathway | 1.99E-05 |

**Table S4. Top enriched terms and biological pathways identified by functional analysis of the DEGs from MERS-CoV .**Overlap: indicates the number of hits from the meta-analysis compared to each curated gene set library. Gene set functional analysis was performed using extended libraries of the EnrichR tool. Enriched terms and pathways were ranked based on the *p-value*. KEGG: Kyoto Encyclopedia of Genes and Genomes ; GO: gene ontology biological process; GSEA: Gene Set Enrichment Analysis.

| **Enrichment Term** | **Pathway/Term ID** | **Overlap** | **GSEA library** | **Adjusted**  **P-value** |
| --- | --- | --- | --- | --- |
| **Insulin Signaling Pathway** | h insulinPathway | 05/115 | Biocarta | 0.187503 |
| **Epithelial cell signaling in Helicobacter pylori infection** | hsa05120 | 14/68 | KEGG | 0.156879 |
| **Apoptosis signaling pathway** | P00006 | 14/102 | Panther | 0.022202 |
| **Unfolded Protein Response** | R-HSA-381119 | 15/86 | Reactome | 0.001967 |
| **Ciliary landscape** | WP4352 | 30/216 | Wikipathway | 0.461864 |

**Table S5. Top enriched terms and biological pathways identified by functional analysis of the DEGs from SARS-CoV .**Overlap: indicates the number of hits from the meta-analysis compared to each curated gene set library. Gene set functional analysis was performed using extended libraries of the EnrichR tool. Enriched terms and pathways were ranked based on the *p-value*. KEGG: Kyoto Encyclopedia of Genes and Genomes ; GO: gene ontology biological process; GSEA: Gene Set Enrichment Analysis.

|  | **Substrates/targets in Input** | **Substrates/targets in Database** | **Input Fraction** | **Database Fraction** | **Difference** | **P-value** | **Z-score** |
| --- | --- | --- | --- | --- | --- | --- | --- |
| **Kinases** | | | | | | | |
| **MAPK1** | 48 | 312 | 0.225352 | 0.068829 | 0.156524 | 1.59E-12 | -2.2592 |
| **MAPK3** | 41 | 251 | 0.192488 | 0.055372 | 0.137117 | 1.74E-11 | -2.22385 |
| **AKT1** | 35 | 256 | 0.164319 | 0.056475 | 0.107845 | 4.30E-08 | -2.24489 |
| **TGFBR1** | 29 | 166 | 0.13615 | 0.03662 | 0.09953 | 6.09E-09 | -2.01002 |
| **MAPK8** | 36 | 275 | 0.169014 | 0.060666 | 0.108348 | 7.32E-08 | -2.06032 |
| **TGFBR2** | 14 | 37 | 0.065728 | 0.008162 | 0.057565 | 2.60E-08 | -1.87941 |
| **MAP3K7** | 21 | 102 | 0.098592 | 0.022502 | 0.07609 | 7.96E-08 | -1.80544 |
| **GSK3B** | 56 | 600 | 0.262911 | 0.132363 | 0.130548 | 5.94E-07 | -1.88477 |
| **HIPK2** | 14 | 43 | 0.065728 | 0.009486 | 0.056242 | 1.22E-07 | -1.52427 |
| **BMPR1B** | 17 | 77 | 0.079812 | 0.016987 | 0.062826 | 6.28E-07 | -1.62827 |
| **Transcription factors** | | | | | | | |
| **SMAD3** | 55 | 1936 | 0.263158 | 0.064525 | 0.198633 | 3.04E-19 | -1.55477 |
| **SMAD2** | 55 | 1936 | 0.263158 | 0.064525 | 0.198633 | 3.04E-19 | -1.55477 |
| **SOX2** | 57 | 2564 | 0.272727 | 0.085455 | 0.187272 | 2.85E-15 | -1.57971 |
| **ESR2** | 17 | 424 | 0.08134 | 0.014131 | 0.067208 | 1.41E-08 | -1.75996 |
| **FOXA2** | 55 | 2968 | 0.263158 | 0.09892 | 0.164238 | 1.37E-11 | -1.04477 |
| **TP53** | 22 | 827 | 0.105263 | 0.027563 | 0.0777 | 1.26E-07 | -1.43918 |
| **GATA1** | 47 | 2601 | 0.22488 | 0.086688 | 0.138192 | 1.41E-09 | -0.93267 |
| **CLOCK** | 14 | 407 | 0.066986 | 0.013565 | 0.053421 | 1.60E-06 | -1.41937 |
| **PAX3-FKHR** | 24 | 1063 | 0.114833 | 0.035429 | 0.079404 | 6.00E-07 | -1.26908 |
| **GATA1** | 18 | 967 | 0.086124 | 0.032229 | 0.053895 | 1.84E-04 | -1.44498 |

**Table S6**- Top ten TFs and Kinases predicted from the DEGs unique to SARS-CoV-2 based on regulatory gene network analysis
